# Pseudo-normalization of a coronary artery aneurysm detected by IVUS

**DOI:** 10.21542/gcsp.2017.32

**Published:** 2017-10-31

**Authors:** Sherif Rizk, Waseem Amin, Hala Hamza, Karim Said, Galal El Said

**Affiliations:** Department of Cardiology, Kasr El Aini Hospital, Faculty of Medicine, Cairo University, Egypt

## Introduction

Coronary artery aneurysm (CAA) is found in 0.3–5% of patients undergoing coronary angiography. Atherosclerosis is the main cause, followed by Kawasaki disease and infectious emboli. The pathogenetic mechanisms underlying aneurysm formation have not been clearly delineated, but inflammation is suspected to play a role. Symptoms, if present, are usually related to myocardial ischemia. In adults, angiography is the mainstay for diagnosis. Management varies from antithrombotic therapy to PCI or surgical treatment^1^.

## Case report

**Figure 1.**
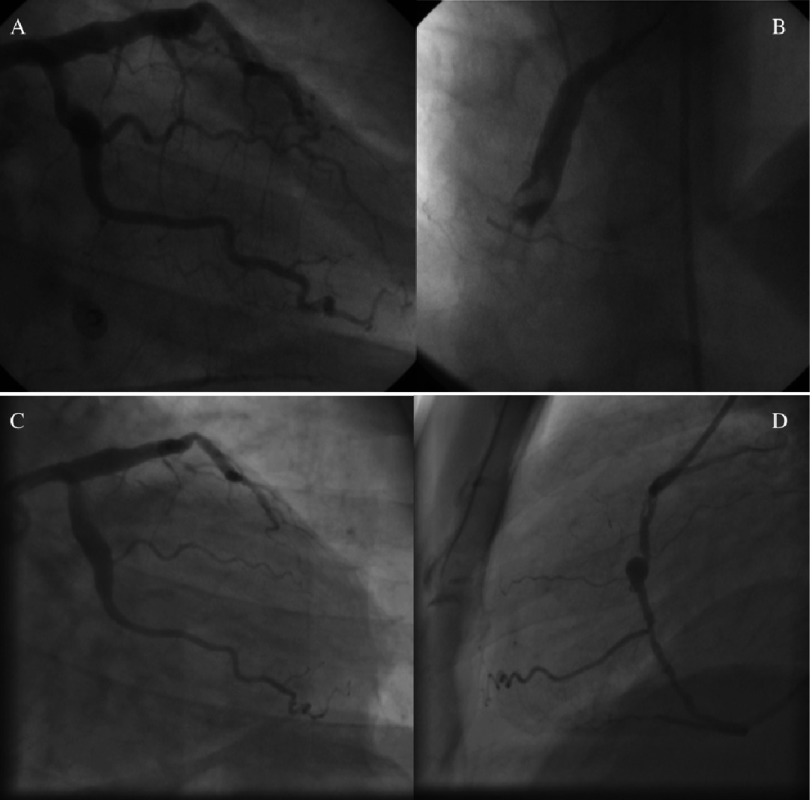
Coronary angiograms at age 40 (A and B) and 44 (C and D) years at the time of presentation with acute coronary syndrome. (A and B) Showing large proximal LAD, LCX and RCA aneurysms with the mid RCA totally occluded with large thrombus. (C and D) Angiogram showing persistence of the LAD and LCX aneurysms with restoration of flow in the distal RCA. The proximal portion of the previous RCA aneurysm is partially occluded with linear filling defects within the lumen presumably representing thrombus.

A 44-year-old man presented with crescendo angina (CCSC III) beginning two weeks before admission. His only cardiovascular risk factor was smoking with a history of Kawasaki disease (KD)-compatible illness during childhood and with no history of diabetes, hypertension or family history of coronary artery disease (CAD). Four years previously, the patient had suffered an infero-posterior ST segment elevation myocardial infarction (STEMI). Coronary angiography at another facility at that time revealed marked aneurysmal dilatation of the proximal segments of the left anterior descending (LAD), left circumflex (LCX), and right coronary (RCA) arteries. The mid-segment of the RCA was totally occluded with a large thrombus; distal branches of RCA filled faintly via retrograde collateral flow from the left coronary system (Figures 1A and 1B). The patient was discharged on warfarin, bisoprolol, atorvastatin, and aspirin. He was compliant for four months and then discontinued all medications and continued to smoke 1 pack of cigarettes per day. He remained asymptomatic until 2 weeks prior to presentation at our center. On admission, cardiovascular examination was unremarkable, the patient’s resting 12-lead electrocardiogram showed the old inferior MI with normal serial cardiac troponin levels and normal laboratory test results including LDL-C level, erythrocyte sedimentation rate, and C-reactive protein.

Echocardiography revealed preserved left ventricular ejection fraction (56%) with akinetic basal and mid segments of the inferior and posterior walls. Coronary angiography revealed significant luminal narrowing of proximal and mid segments of the RCA with a proximal linear filling defect and TIMI III flow to the distal RCA, in addition to persistence of the LAD and LCX aneurysms. Compared to the previous angiogram four years ago, the mid segment of the LAD showed an area of moderate (50%) narrowing with evident angiographic haziness (Figures 1C and 1D). Intra-vascular ultrasound (IVUS) of the 3 vessels using 20 MHz Eagle Eye Platinum catheter (Volcano Corp.) revealed intimal thickening of the walls of the LAD and LCX aneurysms with no evidence of atherosclerosis and maximal lumen diameter of 7.8 and 7.5 mm respectively (Figures 2A and 2B). The mid segment of the LAD aneurysm showed a mildly organized thrombus occupying the vessel lumen and causing 40% minimum lumen area (MLA) stenosis with MLA of 6 mm^2^ (Figure 2A). IVUS of the RCA showed a large aneurysm affecting the proximal and mid segments of the RCA with maximal lumen diameter 8.2 mm and minimally thickened concentric intima at the mid-section of the aneurysm with the proximal and distal parts of the aneurysm partially occluded by large, organized thrombi and segments showing poorly organized thrombi with areas of recanalization and calcification (linear defects by coronary angiography) (Figure 2C).

**Figure 2A.**
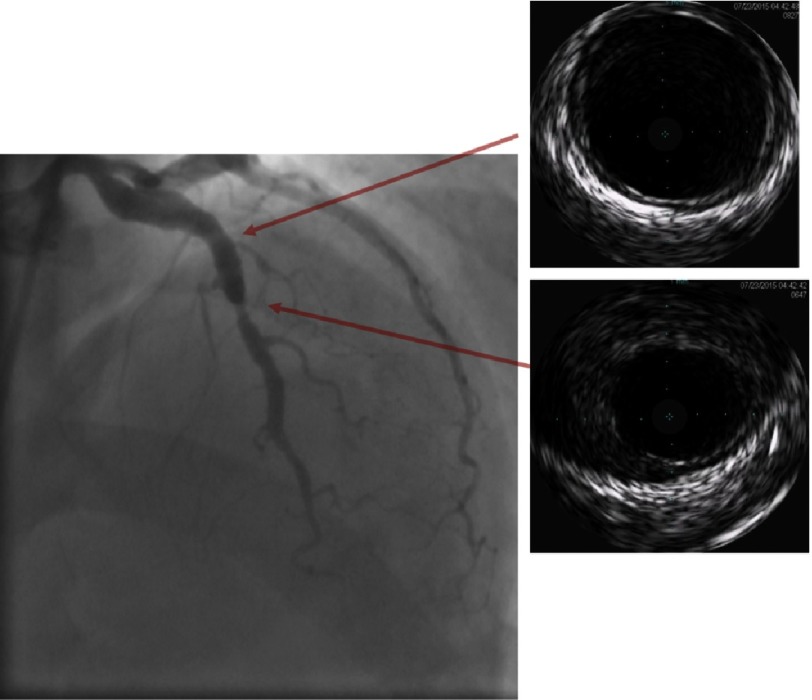
Coronary angiogram with gray-scale IVUS showing proximal LAD aneurysm with moderate stenosis at its outlet with the corresponding IVUS images captured at different locations (red arrows). The mid LAD moderate stenosis is caused by layers of organized thrombi lining the wall and partially occluding the distal part of the LAD aneurysm. There is minimal intimal thickening within the aneurysms with no evidence of atherosclerosis.

**Figure 2B.**
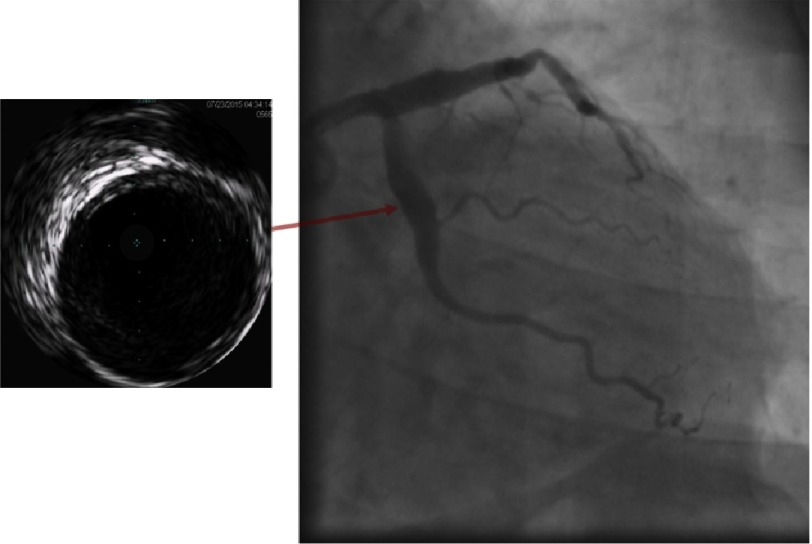
Coronary angiogram with gray-scale IVUS showing proximal LCX aneurysm with the corresponding IVUS image captured at site of the aneurysm showing minimal intimal thickening within the aneurysms and no evidence of atherosclerosis.

**Figure 2C.**
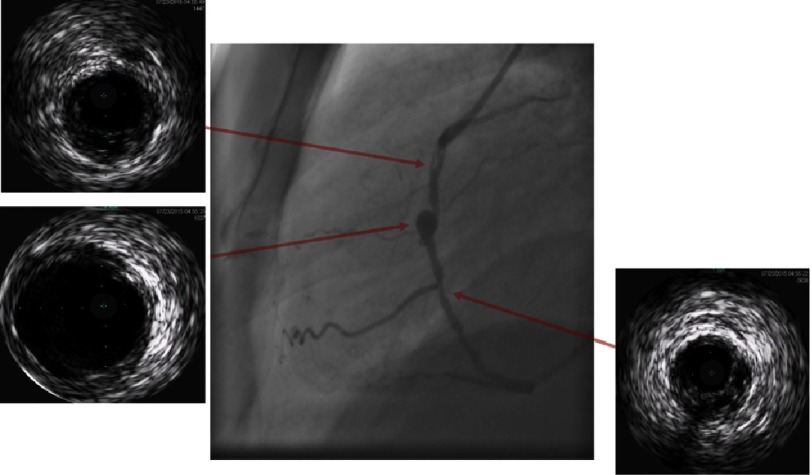
Coronary angiogram with gray-scale IVUS showing mid RCA aneurysm with proximal luminal and linear defects within the lumen of the proximal segment of the RCA with the corresponding IVUS images captured at different vessel locations (red arrows). The mid RCA aneurysm is the remaining patent segment of the aneurysm while proximally and distally, the aneurysm is partially occluded by layers of organized thrombus lining the wall of the aneurysm. The linear defects within the lumen of the RCA are caused by areas of less organized and recanalized thrombi on top of the layers of organized thrombi lining the wall, and encroaching on the lumen partially occluding the proximal and distal segments of the RCA.

There was no evidence of atherosclerosis by false color histology by IVUS. Fractional flow reserve (FFR) measurements using Prestige Pressure Wire (Volcano Corp.) across the mid LAD stenosis with intracoronary adenosine (200 microgram) challenge was 0.86). No interventions were performed and the patient was discharged on warfarin and aspirin with close monitoring of his compliance.

## Discussion

Reports of missed KD in young adults began to emerge following Kawasaki’s original publication and two series, one from Japan and one from the U.S., established that the sequelae of untreated KD in young adults includes myocardial ischemia, infarction, congestive heart failure, and sudden death^2, 3^. Coronary artery aneurysms occur in 25% of untreated KD patients^4^. Recently published guidelines for the evaluation and treatment of adult patients who present with coronary complications of KD are the first attempt to offer strategies for their evaluation and care^5^. However, identifying more precisely a population at risk may have the potential to tailor treatment and improve outcomes^6^.

In a study conducted in Egypt by Rizk et al., 6.7% of young adults who underwent angiography to evaluate symptoms of suspected myocardial ischemia had coronary artery aneurysms that may be due to antecedent KD. This highlights the seriousness and importance of early KD diagnosis and management to avoid its serious cardiovascular sequelae. This is particularly important in Egypt where it seems KD is more common than was anticipated and where the diagnosis is frequently missed^7^.

Coronary artery aneurysm (CAA) identified in the context of acute coronary syndrome (ACS) represents a unique management challenge. CAA is associated with thrombus formation due to disturbed hemodynamics with reduction of the wall shear stress and increased particle resonance time^8^. Once formed, mural thrombus may potentiate the deposition of additional thrombus within the aneurysmal segments. Percutaneous revascularization of CAA has been associated with complications including distal embolization of thrombus, no-reflow phenomenon, stent malapposition, dissection, and rupture^9^.

Angiographic findings that make antecedent KD likely include proximal aneurysms with or without calcification, associated with angiographically normal distal segments^9^. Because a history of KD may be difficult to obtain from young adults who might have been too young to have a personal memory of the illness, recent guidelines from Japan recommend that patients with acute coronary syndromes and aneurysms be diagnosed as having sequelae of KD if other conditions causing aneurysms such as atherosclerosis and collagen vascular disease are excluded^10^.

Our case illustrates the importance of IVUS in determining the underlying cause for luminal narrowing in adult patients with KD coronary complications. Stenosis in chronic phase of KD may be caused by myofibroblast proliferation and intimal hyperplasia or due to thrombus formation within the aneurysms^5, 11^. This makes IVUS a useful tool in preventing interventional complications by revealing organized thrombi, intramural changes and true vessel diameters not specifically detected by conventional coronary angiography. This information is important in providing the optimal management for the patient by preventing undersizing of stents and compression of a large thrombus burden.

Our case illustrates the natural history of coronary aneurysms most probably due to antecedent KF as there was no evidence of typical features of atherosclerosis, such as necrotic cores or lipid pools, identified by IVUS. This is in line with previous histopathological studies in KD autopsies^11, 12^. Our case shows demonstrates the pseudo-normalization of a coronary aneurysm during follow-up. IVUS documented a dynamic state of thrombosis and recanalization, which is in accordance with the study by Suzuki et al. who followed coronary aneurysms with thrombi in 262 cases of KD using magnetic resonance coronary angiography (MRCA)^13^.

There is a growing consensus that systemic anticoagulation and anti-platelet therapy is critical in KD patients with large aneurysms. Although warfarin is the mainstay of anticoagulation therapy, the newer direct thrombin inhibitors and oral Factor Xa inhibitors will likely play a role in the future. The dramatic clinical improvement in our patient following four months of warfarin and aspirin therapy at the time of his initial presentation supports the idea that medical therapy is a reasonable option in the face of a large thrombus burden. The addition of tissue plasminogen activator (tPA) and aggressive anti-platelet therapy with the IIb/IIa inhibitors should also be considered in the acute setting. For resource limited settings, there is benefit from oral anticoagulation plus anti-platelet therapy with a target INR of 2.0-3.0^14^.

Because of the unique therapeutic challenges associated with these lesions, adult cardiologists should be aware that coronary artery aneurysms in young adults may be due to missed KD in childhood and opt to the use of IVUS whenever a coronary artery aneurysm is suspected to guide optimal management.
